# *Metallosphaera sedula* bifurcates into two sizes when it is cultured mixotrophically on soluble iron

**DOI:** 10.3389/fmicb.2025.1455423

**Published:** 2025-05-09

**Authors:** Robert C. Blake, Richard G. Painter, Nghi Pham, Olivia Griswold, Brooke White, Richard A. White

**Affiliations:** ^1^Division of Basic Pharmaceutical Sciences, Xavier University of Louisiana, New Orleans, LA, United States; ^2^NCRC, Department of Bioinformatics and Genomics, University of North Carolina at Charlotte, Kannapolis, NC, United States; ^3^CIPHER, Department of Bioinformatics and Genomics, University of North Carolina at Charlotte, Charlotte, NC, United States; ^4^Australian Centre for Astrobiology, University of New South Wales, Sydney, NSW, Australia

**Keywords:** *Metallosphaera sedula*, integrating cavity absorption meter, *in situ* kinetics, two cell sizes, phenotypic heterogeneity

## Abstract

*Metallosphaera sedula* is a thermoacidophilic archaeon that obtains all of its energy for growth from aerobic respiration and oxidative phosphorylation at the expense of selected organic and inorganic sources of electrons. Initial velocities for the oxidation of soluble ferrous ions by intact cells at 60 °C and pH 1.5 were determined using an integrating cavity absorption meter that permitted accurate absorbance measurements to quantify the increase in soluble ferric iron in the presence of turbid suspensions of the live organisms. *M. sedula* that was cultured on yeast extract either in the absence or the presence of 20 mM soluble ferrous iron exhibited turnover numbers for soluble iron oxidation of 304 ± 26 and 333 ± 31 attamoles/cell/min, respectively. These functional data were consistent with the transcriptomic evidence presented by others, that the proteins presumably responsible for aerobic respiration on soluble iron are expressed constitutively in *M. sedula*. Intact cells of *M. sedula* were characterized by electrical impedance, laser light diffraction, and transmission electron microscopic measurements. All three types of measurements were consistent with the surprising observation that cells cultured on yeast extract in the presence of soluble iron bifurcated into approximately equal numbers of coccoidal cells of two sizes, smaller cells with an average diameter of 0.6 μm and larger cells with an average diameter of 1.35 μm. Cells cultured on the same concentration of yeast extract but in the absence of soluble iron comprised a single cell size with an intermediate average diameter of 1.06 μm. This unexpected bifurcation of a clonal cell population into two demonstrably different sizes when the extracellular nutrient environment changes has not previously been reported for *M. sedula*, or any other single-celled archaeon or eubacterium.

## 1 Introduction

*Metallosphaera sedula* is a thermoacidophilic archaeon in the phylum *Crenarchaeota* that was isolated from an acidic hot water pond at Pisciarelli Sulfatata in Naples, Italy (Huber et al., 1989). Cells are regular to slightly irregular cocci, about 0.6 to 1.5 μm in diameter (Huber and Stetter, 2015). Growth occurs at 50 to 80°C (optimal 65 to 75°C) and pH 1.0 to 6.5 (optimal 2.5 to 3.5). *M. sedula* is aerobic and facultatively chemolithoautotrophic. Heterotrophic growth occurs on complex organic compounds such as yeast extract, casamino acids, peptone and tryptone. Autotrophic or mixotrophic growth occurs in the presence of reduced sulfur compounds, selected sulfide minerals, and soluble ferrous iron. The *Metallosphaera* genus contains six species that are currently recognized (Fuchs et al., 1995; Kurosawa, 2003; Liu et al., 2011; Peng et al., 2015; Kozubal et al., 2011). Other genera within the order *Sulfolobales* that harbor archaea that greatly resemble *M. sedula* include *Acidianus* (5 species) (Segerer et al., 1986; Plumb et al., 2007; Fuchs et al., 1996; He et al., 2004; Yoshida et al., 2006; Urbieta et al., 2017), *Sulfurisphaera* (3 species) (Kurosawa et al., 1998; Suzuki et al., 2002; Tsuboi et al., 2018), *Sulfuracidiflex* (2 species) (Huber and Stetter, 1991; Itoh et al., 2020), and *Sulfodiicoccus* (Sakai and Kurosawa, 2017). All of these thermoacidiphilic archaea are described as lobed or irregular cocci with diameters from 0.5 to 1.8 μm (Liu J. et al., 2021; Liu L. J. et al., 2021; Lewis et al., 2021).

A principal feature of the energy metabolism of these physiologically related *Sulfolobales* is their ability to respire aerobically on soluble reduced iron. Despite the interest in this activity for its contribution to the oxidation and dissolution of minerals within ore bodies, the biomolecules and electron transfer reactions that participate in respiration on soluble iron remain poorly understood. Early studies simply reported the novel spectral properties of presumed electron transfer proteins that were observed in cell-free extracts in archaeal cells that were cultured aerobically on soluble iron (Barr et al., 1990; Blake et al., 1993). A proteomic and transcriptomic study in cell-free extracts of *Sulfuracidiflex* (formerly *Sulfolobus*) *metallicus* first identified proteins and gene products that appeared to be upregulated after the cells were exposed to ferrous iron (Bathe and Norris, 2007). The relevant *fox* genes were subsequently reported from genomic studies to represent terminal heme-copper oxidases whose primary structures were unique to the archaea (Hemp and Gennis, 2008; Sousa et al., 2011). Others have reported further genomic, proteomic, and transcriptomic studies for iron oxidation determinants in the extremely thermoacidophilic archaea (Kozubal et al., 2011; Auernik and Kelly, 2008; Auernik et al., 2008). There is now strong circumstantial evidence that the *fox* gene products, which appear *via* proteomics and transcriptomics to be expressed constitutively in *M. sedula*, are responsible for conducting the electron transfer reactions that comprise aerobic respiration on soluble iron (Counts et al., 2022). An ongoing problem, however, is that accompanying functional assays to quantify iron oxidation by intact cells or cell-free extracts have lagged far behind the elegant genomic, metagenomic, proteomic, and transcriptomic studies. Thus a typical functional measurement consists of a single, fixed-time assay where a sample is removed and treated with 1,10-phenanthroline or a similar organic compound that complexes with ferrous iron and changes color for optical quantification. In order to fully understand and appreciate any biochemical event or reaction, one has to actually monitor the event or reaction as it occurs in real time; simply cataloging the participants, no matter how detailed and thorough is the list, isn't sufficient.

This laboratory has described an integrating cavity absorption meter (ICAM) that permits accurate absorbance measurements to be conducted in turbid media like suspensions of intact cells (Blake and Griff, 2012; Li et al., 2015; Blake et al., 2016; Blake and White, 2020; Blake et al., 2020, 2021). Because ferrous and ferric iron absorb light differently, the ICAM can be exploited to monitor soluble iron oxidation spectrophotometrically as a continuous process. The experimental observations presented below were conducted to quantify the rates of iron oxidation by intact cells of *M. sedula*. In particular, we sought to distinguish between two hypotheses: that the respiratory proteins that are responsible for iron oxidation are expressed constitutively; or that they are induced when the cells are exposed to substrate-level concentrations of soluble iron. Organotrophic and mixotrophic growth on yeast extract in the absence and the presence, respectively, of 20 mM ferrous sulfate produced intact cells that had similar specific activities per intact cell for the rate of iron oxidation. In the process of enumerating and characterizing both populations of cells, we made the unexpected observation that cells cultured in the presence of soluble iron assumed one of two sizes that bracketed the one size assumed by cells cultured in the absence of soluble iron. To our knowledge, no one has reported behavior similar to this in any other iron-oxidizing microorganism, or for that matter, any other microorganism in general.

## 2 Materials and methods

### 2.1 Cell culture

*M. sedula* strain TH2, Deutsche Sammlung von Mikroorganismen und Zellkulturen (DSMZ) 5348^T^, was cultured mixotrophically on 20 mM ferrous sulfate plus 0.2% (wt/vol) yeast extract at 60°C. The pH of the medium was adjusted to 1.5 using sulfuric acid; the minimal salts concentrations were those recommended in the DSMZ media guide for the generic *Sulfolobus* medium, number 88. *M. sedula* was cultured heterotrophically in the same medium that omitted the ferrous sulfate. In either case, cells grown to late stationary phase were harvested by centrifugation, washed twice with 0.02 M H_2_SO_4_, and resuspended in sufficient 0.02 M H_2_SO_4_ to achieve a stock suspension of approximately 1 × 10^10^ cells/mL. Each stock suspension was stored at 4°C for no longer than a week while electrical impedance, spectroscopic, or proteomic measurements were conducted on aliquots of the cells.

### 2.2 Quantification and characterization of microorganisms

Absolute numbers of *M. sedula* cells were determined by electrical impedance measurements in a Multisizer 4 particle counter (Beckman Coulter, Inc., Brea, CA) fitted with a 30-μm aperture (Blake and Griff, 2012; Li et al., 2015; Blake et al., 2020). The instrument was programmed to siphon 50 μL of sample that contained Isoton II as the electrolyte. The current applied across the aperture was 600 μA. Voltage pulses attendant with impedance changes as particles passed through the aperture were monitored with an instrument gain of four. Determinations of particle diameters, surface areas, and volumes were accomplished with operating and analysis software provided by Beckman Coulter, Inc.

Relative numbers of *M. sedula* cells were determined by photon correlation scattering spectroscopy with a DelsaNano C particle size analyzer, also from Beckman Coulter, Inc. Cell densities were adjusted to ~1 × 10^7^ cells/mL in 0.02 M sulfuric acid to give an attenuator obscuration of between 45 and 50%. Determination of the relative numbers of light scattering species as a function of particle diameter was accomplished by the time domain method with operating and analysis software provided by Beckman Coulter, Inc.

Transmission electron microscopy was conducted on a model HT7700 Transmission Electron Microscope from Hitachi High-Tech America, Inc., Schaumburg, IL. Washed cells of *M. sedula* cultured mixotrophically were mixed with 1% (w/v) paraformaldehyde for 30 min at room temperature and then air-dried on a Formvar-coated copper grid for whole mounts.

### 2.3 Initial velocity kinetic measurements

Initial velocity kinetic measurements on the rate of iron oxidation by intact *M. sedula* were obtained by conducting absorbance measurements on the time-dependent appearance of ferric ions using an OLIS-CLARiTY 1000A spectrophotometer (On Line Instrument Systems, Inc., Bogart, GA) that employed a novel integrating cavity absorption meter (Blake and Griff, 2012; Li et al., 2015; Blake et al., 2020). In a typical measurement, identical 8.0- and 7.9-mL solutions that contained sulfuric acid (pH 1.5) were added to both the reference and the sample observation cavities, respectively, of the spectrophotometer. The sample cavity contained an additional 20 μL of a stock cell suspension of cells. After a stable baseline was recorded from 296 to 400 nm, 100 μL that contained soluble ferrous sulfate were added to the cell suspension to initiate subsequent oxidation reactions and absorbance changes within the cavity. These liquid handling operations were conducted manually. The single opening to the otherwise enclosed observation cavity was subsequently fitted with a white, refractive Teflon plug, and data acquisition was then initiated. These latter operations were routinely conducted in 0.5 s, which comprised the operational dead time for any oxidation to proceed before data were obtained.

Raw absorbance spectra, typically 6.2 spectra/s, were collected for appropriate time intervals. Raw absorbance values obtained in the CLARiTY spectrophotometer were converted to equivalent absorbance values/cm using Fry's method (Fry et al., 2010) with analysis software provided by OLIS, Inc. Global fits of absorbance changes as a function of both time and wavelength were accomplished by the singular value decomposition method (DeSa and Matheson, 2004) using analysis software also provided by OLIS, Inc.

### 2.4 Ribosomal RNA sequence analyses

Sequence analyses of 16S ribosomal RNA were conducted by the Zymo Research Corporation, Irvine, CA. Cells of *M. sedula* that were cultured to stationary phase either heterotrophically or mixotrophically were harvested by centrifugation, washed twice with distilled water to remove excess acid and soluble iron, and resuspended and frozen in sufficient distilled water to achieve samples comprised of 200 mg wet-weight cells in 1.8 mL. Briefly, Zymo Research then extracted the DNA from both samples, prepared the DNA for targeted sequencing using the Quick-16S^TM^ Primer Set, and sequenced the resulting libraries using an Illumina NextSeq2000^TM^ (Illumina, San Diego, CA). Subsequent bioinformatics analyses were conducted using the Dada2 pipeline (Callahan et al., 2016) that was supplemented with the sequence information for *M. sedula* DSMZ 5348^T^.

### 2.5 Scanning electron microscopy and energy dispersive x-ray analyses

Scanning electron microscopy (SEM) and Energy dispersive x-ray spectroscopy (EDS) measurements were conducted by the Drug Discovery and Delivery Core facility at Xavier University of Louisiana, New Orleans, LA. Cells of *M. sedula* that were cultured to stationary phase either heterotrophically or mixotrophically were harvested by centrifugation, washed twice with distilled water to remove excess acid and soluble iron, and resuspended in sufficient distilled water to achieve samples comprised of 900 mg wet-weight cells in 15 mL. Briefly, 20 μL of washed, suspended cells were deposited on the surface of copper tape that was affixed to the sample stub that was positioned in the analysis chamber of a model S-3400N SEM (Hitachi High-Tech America, Inc., Schaumburg, IL) that was equipped with a model EDAX Elite T EDS system (Ametek, Pleasanton, CA). The deposited cells were air-dried and analyzed in a vacuum.

## 3 Results

### 3.1 Quantification and size characterization of archaeal cells

**Figure 1:**
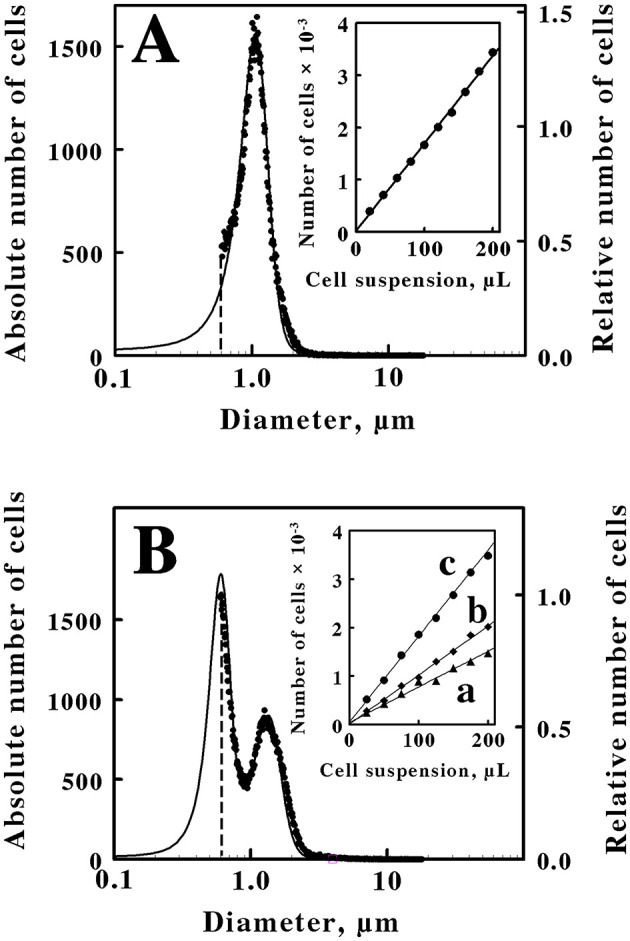
Quantification and characterization of intact *M. sedula* cultured heterotrophically on yeast extract **(A)** or mixotrophically on yeast extract plus 20 mM ferrous sulfate **(B)**. Each panel compares cell suspensions by electrical impedance (*data points, left ordinate*) and static light diffraction (*solid curve, right ordinate*) measurement methods. The *dashed vertical lines* in **(A, B)** represent the lower limits of detection for the electrical impedance method. The *insets* in **(A, B)** show the numbers of cells as a function of the microliters of the cell suspension that were derived from the heterotrophic and the mixotrophic cultures, respectively. *Curves a, b*, and *c* in the *inset* of **(B)** represent cell counts for cell diameters of 0.6 to 0.95 μm, 0.95 to 3.0 μm, and 0.6 to 3.0 μm, respectively. Each *datum* in the *insets* of **(A, B)** represents the mean of 6 and 10 determinations, respectively; in all cases, the corresponding standard deviations are within the diameters of the data points.

Figure 1A shows the size and quantitative measurements that were conducted on cells of *M. sedula* that were cultured heterotrophically on just yeast extract. These measurements were conducted using two methods, electrical impedance and static light scattering. The results from the two methods agreed that the coccoidal cells comprised a single population with an average diameter of 1.06 μm. It must be noted that each method has strengths and weaknesses. The strength of the electrical impedance measurement is that it supplies absolute numbers for the different cell sizes in the culture; the weakness is that the coccoidal archaea with spherical diameters <0.6 μm were underrepresented by the electrical impedance measurements, by which observations were limited to particles with effective diameters between 2 and 60% of the 30-μm aperture employed in the Multisizer. The strength of the static light diffraction measurements is that they are capable of resolving particles with effective diameters as small as 0.1 nm; the weakness is that the light diffraction method does not yield absolute numbers of cells, only relative numbers of cells in the entire population. However, the two types of measurements, when used in conjunction, serve to complement each other to provide a more complete characterization of the entire cell populations.

In the present case, the immediate goal was to quantify the rate of iron oxidation by intact cells. Consequently, the desire was to accurately determine the absolute number of cells to calculate an accurate turnover number per cell in the intact cell-dependent catalytic activity assays. The *inset* in Figure 1A shows the standard curve that was developed to quantify the number of cells present in a stock suspension of washed cells that were derived from the heterotrophic culture of *M. sedula* on yeast extract. Different volumes of the stock suspension were removed and added to the electrolyte solution that was employed in the Multisizer. The numbers of the resulting cells that were present in a prescribed volume of the electrolyte-cell mixture were then plotted as a function of the volumes of the aliquots that were removed from the stock suspension. The number of cells in each aliquot was directly proportional to the volume of the aliquot that was withdrawn from the stock suspension. This standard curve provided the means to quantify the number of cells present in any aliquot taken from the stock suspension for use in subsequent kinetic measurements. It is evident from the data in Figure 1A that a small number of the total cells in the heterotrophic suspension were not counted due to the sensitivity limitation of the electrical impedance measurements. Fortunately, a close correspondence between the electrical impedance and the light diffraction curves was observed down to a diameter of 0.6 μm, indicating that the two instruments were monitoring the same population of particles. Consequently, the numbers of cells that were actually counted by the electrical impedance measurements were increased by 11% to account for the portion of the light diffraction measurements that fell below the limit of detection of the absolute counting method.

Figure 1B shows the size and quantitative measurements that were conducted on cells of *M. sedula* that were cultured mixotrophically on the same concentration of yeast extract amended with 20 mM ferrous sulfate. In this case, the resulting cells comprised two populations of coccoidal cells with different average diameters of 0.6 and 1.35 μm. Once again, a close correspondence between the electrical impedance and the light diffraction curves was observed down to a diameter of 0.6 μm, indicating that the two instruments were monitoring the same population of particles. The *inset* in Figure 1B shows the standard curve that was developed to quantify the numbers and types of cell sizes present in a stock suspension of washed cells that were derived from the mixotrophic culture of *M. sedula*. *Curve c* represents the standard curve for the total number of cells that were quantified in each aliquot. Because the mixotrophic population of cells consisted of two different sizes, a cell diameter of 0.95 μm was arbitrarily chosen as the dividing diameter between the two different cell sizes in the bifurcated mixture. *Curves a* and *b* in the *inset* of Figure 1B thus represent the standard curves for the smaller (0.6 to 0.95 μm cell diameters) and the larger (0.95 to 3.0 μm cell diameters) cells, respectively, within the bifurcated mixture. Although the actual cell counts in the *inset* indicate that the larger cell cohort outnumbered the smaller cell cohort, it is evident that the two curves overlap to a considerable extent. It is also evident in the *main panel* that approximately half of the smaller diameter cells were not quantified by the electrical impedance method. The relative contributions of the two different cell sizes that are evident in Figure 1B to the overall number of cells in the bifurcated mixotrophic cultures were estimated by iterative nonlinear regression analyses of the electrical impedance and the light diffraction measurements (calculations not shown). The result of these calculations was that the small and large cell cohorts contributed ~48 and 52 percent, respectively, to the total cell numbers in the mixotrophic culture. That is, the mixotrophic culture consisted of approximately equal numbers of the two cell sizes.

**Figure 2:**
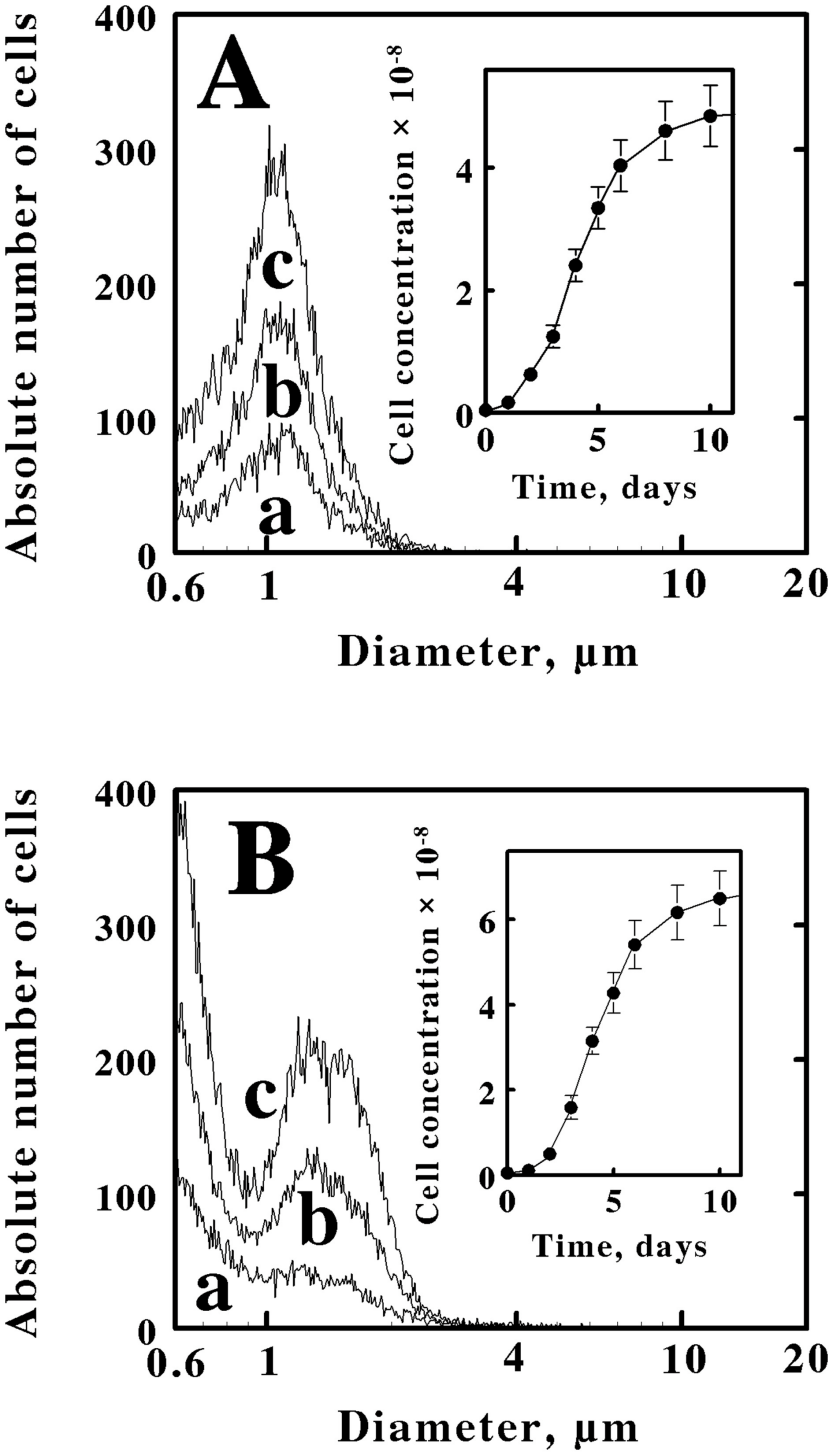
Electrical impedance measurements of the time dependencies and cell size characteristics of the cells that were obtained when *M. sedula* was inoculated into different media. **(A)** 6.1 × 10^6^ cells of stationary phase *M. sedula* that were cultured mixotrophically in yeast extract plus soluble ferrous iron were inoculated into fresh media that contained only yeast extract. **(B)** 5.3 × 10^6^ cells of stationary phase *M. sedula* that were cultured heterotrophically in yeast extract were inoculated into fresh media that contained yeast extract amended with soluble ferrous iron. *Curves a, b*, and *c* in both panels represent the cell populations present at 3, 4, and 6 days, respectively. The cell numbers in both *insets* represent the concentrations of cells present in the culture at each corresponding time period. Each *datum* in both *insets* represents the mean of 8 determinations; in some cases the corresponding standard deviations are within the diameters of the data points.

An alternative hypothesis for the particles that comprise the small sized fraction in Figure 1B is that these smaller particles actually represent exosomes that are produced by *M. sedula* when soluble iron is added to the yeast extract medium. Exosomes, or extracellular vesicles, are membrane-bound particles of variable diameter that are excreted into the extracellular milieu by all three domains of life (Liu J. et al., 2021; Liu L. J. et al., 2021). *M. sedula* does produce biogeochemically active archaeal membrane vesicles (Johnson et al., 2018). However, energy deprivation was evaluated in *M. sedula* and found to stimulate vesicle synthesis, while energy excess repressed vesicle formation. Thus autotrophic culture on low energy-yielding ferrous sulfate supported vesicle synthesis, while the addition of an organic carbon and energy source (in their case, tryptone) prevented vesicle synthesis as indicated by microscopy and protein quantification. Furthermore, the mean diameter of the vesicles that were produced under autotrophic conditions on soluble iron was only 194 ± 36 nm. The light diffraction measurements in Figures 1A, B show no hint of a population of particles in our cultures that were smaller than a population with a mean diameter of 600 nm. Based on both size and culture condition comparisons, we conclude that the smaller particles in Figures 1B, 2B do not represent extracellular exosomes.

These observations that the same cells could assume one of two cell-size profiles, depending on the medium in which the cells were cultured, were reproducible. Figure 2A shows three representative profiles of cell counts as a function of cell diameters that were obtained when a limited number of cells of M. *sedula* that were cultured mixotrophically were inoculated into fresh media that only contained yeast extract. All three curves were consistent with the pattern observed with cells cultured heterotrophically, like those presented in Figure 1A, where the cell counts were dominated by coccoidal cells that comprised a single population with an average diameter of 1.06 μm. There was no hint of the two different cell sizes that are evident in the culture shown in Figure 1B. The *inset* in Figure 2A shows the corresponding growth curve for *M. sedula* that was taken from a mixotrophic culture and grown in the heterotrophic medium without amended soluble ferrous iron. The cell counts on the *ordinate axis* of the *inset* represent the actual total cell counts obtained when 10 μL of the culture were added to 10 mL of Isoton, and 50 μL of the resulting mixture were subsequently aspirated through the measuring aperture in the instrument. After 14 days, the heterotrophic culture achieved a stationary phase that contained 5.4 × 10^8^ cells/mL.

Figure 2B shows three representative profiles of cell counts as a function of cell diameters that were obtained when a limited number of cells of M. *sedula* that were cultured heterotrophically were inoculated into fresh media that contained yeast extract amended with 20 mM ferrous sulfate. All three curves were consistent with the pattern observed with cells cultured mixotrophically, like those presented in Figure 1B, where the cell counts were dominated by coccoidal cells that comprised two populations of coccoidal cells with different average diameters of 0.6 and 1.35 μm. There was no hint of the single cell size that is evident in the culture shown in Figure 1A. The *inset* in Figure 2B shows the corresponding growth curve for *M. sedula* that was taken from a heterotrophic culture and grown in the mixotrophic medium amended with soluble ferrous iron. After 14 days, the culture had achieved a stationary phase that contained 7.6 × 10^8^ cells/mL.

**Figure 3:**
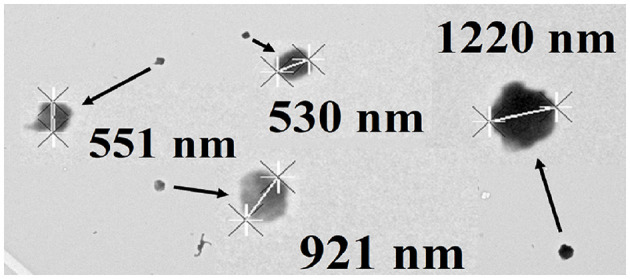
Transmission electron micrograph of *Metallosphaera sedula* cultured mixotrophically on yeast extract and 20 mM soluble ferrous ions at pH 1.5. Four coccoid cells of representative sizes are featured. Cell diameters were determined using the “point to point” linear measurement method.

Independent testimonial evidence that *M. sedula* bifurcates into two cell sizes when it is cultured mixotrophically was obtained by transmission electron microscopy of cells cultured on yeast extract and soluble iron. Figure 3 shows an example of a transmission electron micrograph that contains four coccoidal cells of *M. sedula* that had been cultured mixotrophically. Two of the individual coccoidal cells exhibited diameters <0.6 μm and thus represented examples of the cohort of small cells. One cell had a diameter of 1.22 μm and thus represented an example of the cohort of large cells. Finally, the remaining cell had a diameter of 0.921 μm and thus could represent either type of cell in the bifurcated mixture.

### 3.2 16S ribosomal RNA sequence analyses

Cells that were cultured either heterotrophically or mixotrophically were subjected to 16S ribosomal RNA sequence analyses to address the hypothesis that our laboratory cultures of *M. sedula* were contaminated with one or more different microorganisms. The 16S rRNA macromolecule is an important structural and functional component of both the bacterial and archaeal 30S ribosomal subunits. Because its primary structure is so highly conserved among species of bacteria and archaea, the 16S rRNA gene has become an important phylogenetic marker, and its sequence is routinely exploited for classification and identification of microorganisms (Woese and Fox, 1977; Woese, 1987; Woese et al., 1990). We sought to test whether changing the culture medium simply permitted one or more members of a hypothetically mixed culture to thrive at the expense of the other members. The sequencing studies revealed that 99.5% of the sequences of the samples cultured in both media were identical to that of *M. sedula* DSM 5348^1^ in the database used for the analyses. The remaining 0.5% of the sequences were so low abundance that they were deemed to represent background noise.

### 3.3 Energy dispersive x-ray spectroscopy

Cells that were cultured either heterotrophically or mixotrophically were subjected to SEM and EDS analyses to address the hypothesis that the particles that are designated as ‘small cells' were actually a relatively uniform collection of smaller particles that included flocculated ferric oxy-hydroxide (FeOOH) and sulfate precipitates. Supplementary Figures S1A, B show energy dispersive x-ray spectrographs for washed cells of *M. sedula* that were cultured to stationary phase at pH 1.5 in the absence (Supplementary Figure S1A) and presence (Supplementary Figure S1B), respectively, of 20 mM soluble iron. In both examples, the principal elements that stood out in the elemental analyses were carbon (1 major peak) and copper (3 peaks). The cell samples were deposited and dried on copper tape, so copper could not help but be prominent in the analyses. In either case, there were no discernible peaks associated with either iron or sulfur. Supplementary Figure S1C shows the spectrograph that was obtained for the washed cells that were cultured mixotrophically in 40 mM soluble iron at pH 3.5. In addition to carbon and copper, the spectrograph in Supplementary Figure S1C also exhibited discrete elemental peaks that were associated with iron and sulfur. The combination of a higher concentration of soluble iron and a higher pH value would greatly encourage the coprecipitation of ferric oxy-hydroxide and sulfate. When examined by the electrical impedance and light diffraction methods employed herein, the resulting profile of cell counts *vs*. particle diameters for cells cultured at pH 3.5 had much lower signal-to-noise characteristics than those profiles shown in Supplementary Figures 1B, 2B (data not shown). Given that the numbers associated with the smaller fraction in Figure 1B were comparable to the numbers associated with the larger fraction, we conclude that the absence of discernible iron and sulfur in the EDS spectrograph in Supplementary Figure S1B is consistent with the conclusion that the smaller fraction in Figure 1B is predominantly carbon-based and not inorganic.

### 3.4 Initial velocity kinetic studies

**Figure 4:**
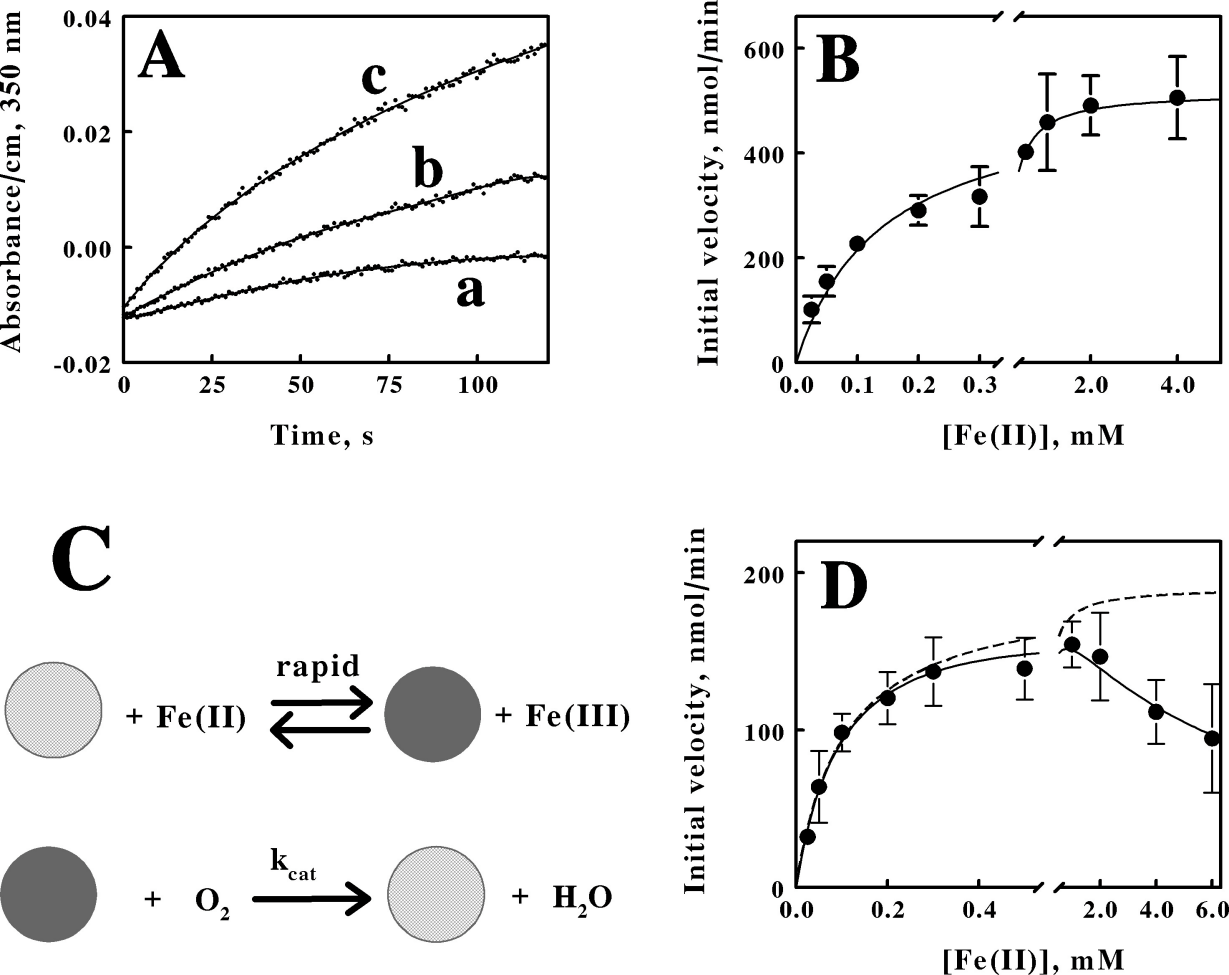
Kinetic behavior of aerobic respiration on soluble iron by intact *M. sedula*. **(A)** *curves a, b*, and *c* are representative examples of the increases in the absorbance at 350 nm when *M. sedula* was mixed with 0.025, 0.1, and 4.0 mM soluble ferrous ions, respectively. The parameters for the *curves* drawn through the *data points* were determined by fitting a sextic polynomial to each set of data. **(B)** dependence of the initial velocity of ferric ion production on the concentration of ferrous ions when 1.7 × 10^9^ cells of heterotrophically-grown *M. sedula* were included in 8 mL of sulfuric acid (pH 1.5) at 60°C. The parameters for the *curve* drawn through the data points were determined by a nonlinear regression analysis using the Michaelis-Menten equation. **(C)** schematic representation of the kinetic mechanism for aerobic respiration on soluble iron as catalyzed by intact *M. sedula*. The *cross-hatched* and *solid spheres* represent archaea that contain oxidized and iron-reduced electron transport proteins, respectively. **(D)** dependence of the initial velocity of ferric ion production on the concentration of ferrous ions when 5.7 × 10^8^ cells of mixotrophically-grown *M. sedula* were included in 8 mL of sulfuric acid (pH 1.5) at 60°C. The parameters for the *solid curve* drawn through the data points were determined by a nonlinear regression analysis using the Michaelis-Menten equation amended to include substrate inhibition. Each *datum* in **(B, D)** represents the mean and standard deviation of 4 determinations. The parameters for the *dashed curve* omitted the term in the equation that represented the effect of the substrate inhibition.

The formation of product ferric ions was evident as soon as intact cells of *M. sedula* were introduced into an aerobic solution of ferrous ions in sulfuric acid, pH 1.5. Figure 4A shows three representative time courses for the increases in absorbance at 350 nm that were obtained when 1.7 × 10^9^ cells of *M. sedula* that were cultured heterotrophically were mixed with different concentrations of ferrous ions and monitored over time at 60°C. We determined an absorption coefficient of 740 M^−1^cm^−1^ at 350 nm for oxidized iron in sulfuric acid, pH 1.5 (see the standard curve in Supplementary Figure S1). Initial velocities of the changes in ferric concentration as a function of time were obtained from primary data such as those shown in Figure 4A, and the resulting secondary plot of initial velocity as a function of the starting ferrous ion concentration in shown in Figure 4B. The parameters for the rectangular hyperbola drawn through the *data points* in Figure 4B were derived from a nonlinear least-squares fit of the initial velocity data to the Michaelis-Menten equation. Values for K_M_ and V_max_ of 140 ± 15 μM and 516 ± 44 nmoles/min, respectively, were obtained from the analysis. That value of V_max_ corresponds to a turnover number of 304 ± 26 attamoles/min/cell.

The schematic illustration shown in Figure 4C represents the minimal kinetic mechanism that is consistent with the initial velocity kinetic data shown in Figure 4B. The iron-dependent reduction of the relevant electron transport proteins in the aerobic respiratory chain in the archaeon is depicted as a relatively rapid reaction, which is consistent with observations that reduction of a colored component within the intact cell is complete within the 0.5 to 1.0 s dead time of the mixing in that instrument (Blake and Griff, 2012; Li et al., 2015; Blake et al., 2016; Blake and White, 2020; Blake et al., 2020). The archaeon with its reduced cellular component(s) then reacts with molecular oxygen to regenerate the oxidized archaeon in a slower reaction that constitutes the rate-limiting catalytic step. When the concentration of intact *M. sedula* cells was limited to that of a catalyst compared with the concentrations of reduced iron and molecular oxygen, aerobic respiration on soluble iron proceeds kinetically in a manner that was consistent with the Michaelis-Menten formalism.

Time courses analogous to those shown in Figure 4A were also obtained when 5.7 × 10^8^ cells of *M. sedula* that were cultured mixotrophically were mixed with different concentrations of ferrous ions and monitored over time at 60°C (primary data not shown). The resulting secondary plot of initial velocities vs. the concentration of reduced iron is shown in Figure 4D. These intact cells also exhibited kinetic behavior that was consistent with the Michaelis-Menten formalism, but with an added deviation. The initial velocity of iron oxidation increased with increasing concentrations of soluble iron up to a point, but the initial velocity then began to decline in value as the concentration of soluble iron increased even further. The *solid curve* drawn through the *data points* in Figure 4D represents the nonlinear regression fit of the data to a velocity equation that represents substrate inhibition (Reed et al., 2010):


(1) $$\begin{array}{l} v_{0}=V_{\max}\;\left(S\right)/\left(K_{M}+\left(S\right)+\frac{{\left(S\right)}^{2}}{K_{I}}\right) \end{array}$$

where v_0_ is the initial velocity and K_I_ is the equilibrium dissociation constant for the substrate inhibition. Values for K_M_, V_max_, and K_I_ of 104 ± 13 μM, 190 ± 18 nmoles/min, and 6.3 ± 1.6 mM, respectively, were obtained from the analysis. The *dashed curve* in Figure 4D represents the initial velocity curve that would have been obtained had there been no substrate inhibition. The uninhibited maximum velocity of 190 nmoles/min corresponds to a turnover number of 333 ± 31 attamoles/cell/min. Interestingly, depending on the degree of substrate inhibition that might occur in nature, the effective turnover number per cell is essentially unaffected by whether soluble iron was present at substrate level concentrations in the growth medium. Consequently, the kinetic data are consistent with the hypothesis that the ability to respire aerobically on soluble iron is expressed constitutively in *M. sedula*.

## 4 Discussion

The original impetus for these studies was to distinguish between two hypotheses: that the respiratory proteins that are responsible for iron oxidation are expressed constitutively; or that the respiratory proteins that are responsible for iron oxidation are induced when the cells are exposed to substrate-level concentrations of soluble iron. The turnover numbers for respiratory iron oxidation that were determined for cells of *M. sedula* that were cultured in the presence or the absence of soluble reduced iron were within experimental error of each other, an observation that is consistent with the hypothesis that the respiratory proteins that are responsible for iron oxidation are expressed constitutively. Determination of these turnover numbers necessarily required that the intact cells that comprised the catalytic units in these studies had to be quantified with as much accuracy as was possible. The efforts to quantify the intact cells led to the unexpected observations regarding cell sizes that comprise the bulk of the studies presented herein.

Planktonic microorganisms that are cultured from a single clone exhibit a narrow range of sizes and volumes, ranging from smaller, newly-formed “daughter” cells to larger cells that are ready to divide into two daughter cells. This range is typically represented by a log-normal distribution when the cell numbers are plotted as a function of the computed diameter of the cell. That is the behavior that we observed when *M. sedula* was cultured heterotrophically on yeast extract (Figures 1A, 2A). It is generally reported that populations of certain microorganisms can adopt different sizes (Taheri-Araghi et al., 2015; Westfall and Levin, 2017) and even shapes (Young, 2006) when they are cultured on different substrates. The average volume of individual cells within a clonal population generally increases in those cells that are exposed to richer nutrients in the environment or decreases if the cells are exposed to fewer nutrients (Harris and Theriot, 2016, 2018; Taheri-Araghi et al., 2015; Westfall and Levin, 2017). However, that generality applies to the entire population of cells, not to a subset of the population. When *M. sedula* was cultured mixotrophically on yeast extract plus 20 mM ferrous ions, which represents a richer nutritional environment than just yeast extract alone, a reasonable hypothesis was that the average volume of the *M. sedula* cells might increase somewhat. Instead, the population of cells bifurcated into two sizes, one larger and one smaller than that obtained when the cells were cultured on yeast extract in the absence of soluble iron (Figures 1B, 2B, 3). There is little, if any, precedent in the microbiological literature for this surprising observation.

One hypothesis that might account for the two cell sizes that appear when *M. sedula* is cultured under a particular set of solution conditions is that the culture of *M. sedula* is actually contaminated with one or more other microorganisms. Over forty years ago, several laboratories reported that selected cultures of mesophilic, iron-oxidizing, acidophilic eubacteria, known collectively as “*Thiobacillus*” or “*Ferrobacillus*” *ferrooxidans*, could be cultured in successive media that alternated between strictly autotrophic soluble ferrous ions and strictly heterotrophic glucose or some related organic substrate (Shafia et al., 1972; Tabita and Lundgren, 1971; Tuovinen and Nicholas, 1977). In each case, the origins of these growth characteristics were traced to the presence of acidophilic, heterotrophic microorganisms that contaminated the autotrophic iron-oxidizing cultures (Harrison et al., 1980; Johnson and Kelso, 1983; Lobos et al., 1986). It was postulated that the heterotrophic contaminant could survive on a sparce diet of organic materials that either “leaked” from or were transported out of the chemolithotroph that was fixing carbon dioxide into cellular material. So is that sort of scenario an explanation for the unexpected culture behavior reported herein? Was the culture of *M. sedula* in our laboratory contaminated with a slightly smaller thermoacidophilic, coccoidal microorganism whose numbers increased to rival those of the *M. sedula* under certain culture conditions? That hypothesis was not consistent with the 16S rRNA sequencing studies summarized above.

Once we accept the premise that clonal *M. sedula* does indeed bifurcate into roughly equal numbers of two cell sizes when soluble ferrous ions are added to complex organic nutrients in the external medium, then the next question becomes, “Why?” Any rationalization for this unexpected behavior is more speculative at this point than hypothesis-driven. The appearance and existence of a large cell size cohort in a culture medium that was amended with additional nutrients (soluble ferrous ions, in this case) is entirely consistent with published observations conducted on numerous other microorganisms in numerous different culture media. The appearance and existence of a small cell size cohort under the same conditions is not. Where would *M. sedula* encounter high concentrations of soluble reduced iron in its natural habitat? Probably in the close proximity of iron-bearing reduced metal-sulfide minerals such as pyrite, marcasite, arsenopyrite, chalcopyrite, *etc*. Would there be a selective advantage to becoming smaller in the presence of electron-rich solid minerals that could serve as a source of electrons to support aerobic respiration? Perhaps. Unpolished minerals rarely present smooth surfaces at their interfaces with liquid media. Rather, minerals in the rough present irregular surfaces that contain numerous small crevices, cracks, invaginations, and so on. A smaller coccoidal microorganism would enjoy greater access to a larger percentage of the irregular surface area of the uneven mineral than would a larger coccoidal microorganism. The greater the physical contact between the surface of the microorganism and the surface of the rough, semi-conducting mineral solid, the greater is the opportunity for direct electron transfer from one entity to the other. Alternatively, if all of the mineral oxidation occurs *via* an indirect mechanism where the soluble iron serves as the mobile agent to conduct electrons from the surface of the mineral to the plasma membrane of the *M. sedula*, then maximizing the microorganism's proximity to the uneven mineral surface by becoming smaller is still a selective advantage because fewer soluble ferrous ions have the opportunity to diffuse away from the nearby microorganism and be “lost.” So why would only a portion of the entire population of planktonic cells transform into a smaller size when the entire population encountered high levels of soluble iron in the medium?

The advent over the last 20 years of methods for quantifying gene expression in individual microbial cells has revealed that populations of genetically identical cells derived from a single clone nonetheless exhibit phenotypic heterogeneity where certain genes are expressed in a non-uniform manner within the population (Ackermann, 2015; Lidstrom and Konopka, 2010; Martins and Locke, 2015; Roberfroid et al., 2016). Consequently, if extracellular environmental conditions change abruptly and unpredictably, at least a small portion of the isogenic population is already pre-adapted and poised to cope with some aspect of the ever-fluctuating environment. On the one hand, this phenotypic heterogeneity may put some or many cells at a disadvantage because they have expressed proteins and capabilities that remain unused and are thus a waste of precious resources at that moment. On the other hand, this practice also means that at least a small portion of the population of cells are in a better position to respond rapidly to the changing conditions and thereby perpetuate the species. “Bistability” is a term that has been used to represent this phenomenon of the bifurcation of an isogenic population of cells into distinct subpopulations (Dubnau and Losick, 2006).

We hypothesize that the bifurcation of *M. sedula* into two cell sizes is an example of phenotypic heterogeneity that is triggered by exposure of the cells to higher concentrations of soluble ferrous ions. If higher concentrations of soluble iron are indeed harbingers of impending proximity to a rich source of electrons derived from semi-conducting, iron-bearing mineral sulfides with uneven surfaces, then that prospect represents a selective advantage to promote the adaptation of the cells to a smaller size. Interestingly, we have observed that *Sulfobacillus thermosulfidooxidans*, a Gram-negative, moderately thermophilic eubacterium, also appears to exist in two different sizes when it is cultured mixotrophically on yeast extract and soluble iron (unpublished data). We hypothesize that populations of other iron-oxidizing microorganisms that can grow both mixotrophically and heterotrophically may also exhibit analogous size differences when they are cultured on organic media in the absence and presence of soluble iron. Perhaps such size differences exist in many iron-oxidizing acidophiles, but they have heretofore escaped notice because researchers have not specifically looked for them. It will be interesting to see whether other examples of this unexpected behavior appear in the future.

## Data Availability

The datasets presented in this study can be found in online repositories. The names of the repository/repositories and accession number(s) can be found here: https://massive.ucsd.edu/ProteoSAFe/dataset.jsp?task=d034f9f6fddc4877bf305c7479181017, MSV000095146, and https://osf.io/4v62d/.

## Footnotes

^1^ https://www.ncbi.nlm.nih.gov/gene/91756283
