# Supplementary material for: Metallosphaera sedula bifurcates into two sizes when it is cultured mixotrophically on soluble iron
Source: Front Microbiol. 2025 May 9;16:1455423. doi: 10.3389/fmicb.2025.1455423 (PMC12101473; doi:10.3389/fmicb.2025.1455423)
Supplement: Supplementary file 1 [file Data_Sheet_1.pdf]

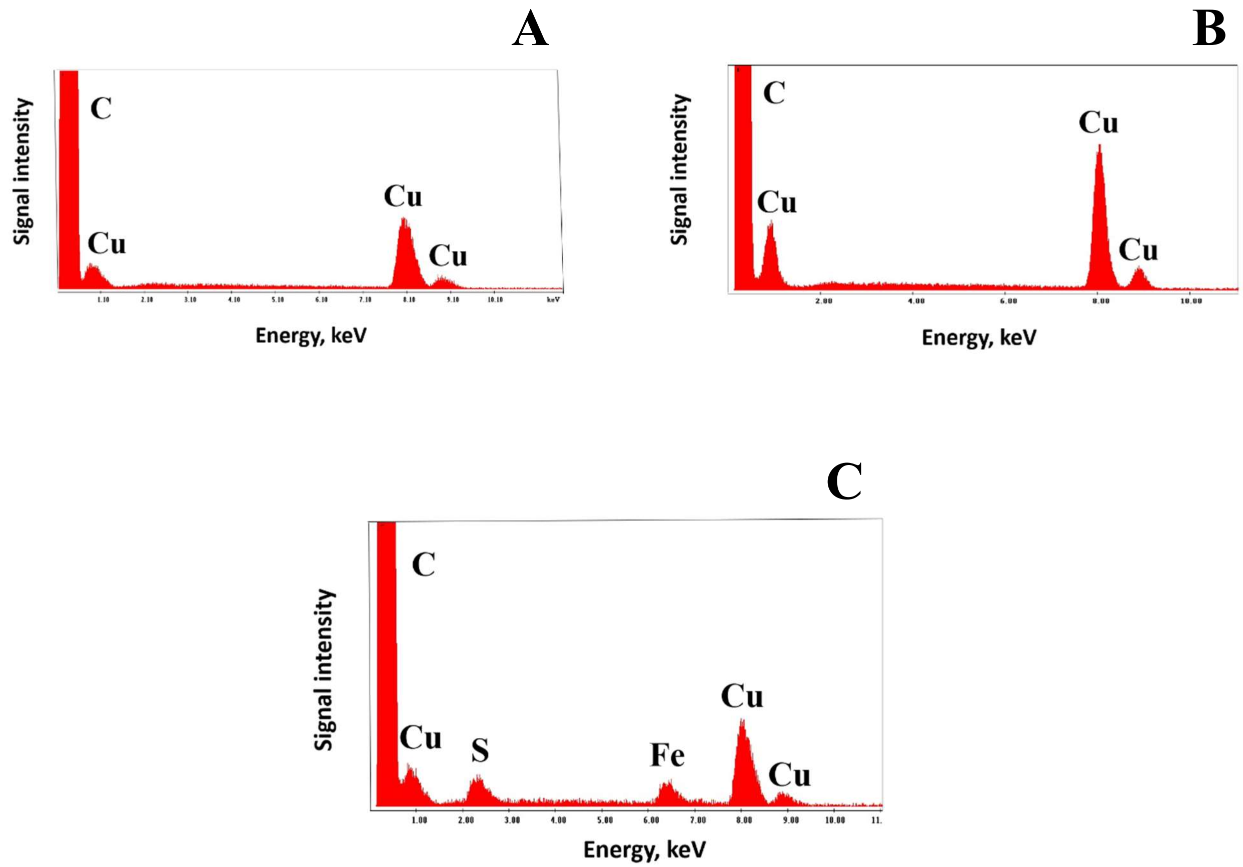

Figure S1. Energy dispersive x-ray spectrographs of samples of *Metallosphaera sedula* that were cultured heterotrophically on yeast extract at pH 1.5 (**A**), mixotrophically on yeast extract amended with 20 mM soluble iron at pH 1.5 (**B**), and mixotrophically on yeast extract amended with 40 mM soluble iron at pH 3.5 (**C**). Elements: C, carbon; Cu, copper; Fe, iron; and S, sulfur.

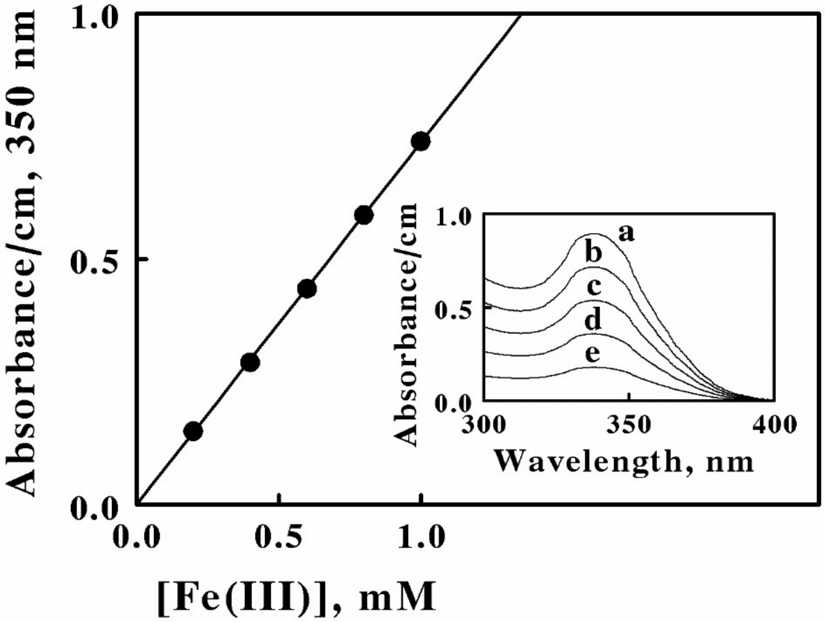

Figure S2. Dependence of the absorbance at 350 nm on the concentration of ferric sulfate at pH 1.5. *Inset*, absorbance spectra of ferric sulfate at pH 1.5; ferric concentrations: 0.2, 0.4, 0.6, 0.8, and 1.0 mM in curves *a* through *e*, respectively.
